# Development of Thin Film Microextraction with Natural Deep Eutectic Solvents as ‘Eutectosorbents’ for Preconcentration of Popular Sweeteners and Preservatives from Functional Beverages and Flavoured Waters

**DOI:** 10.3390/molecules29194573

**Published:** 2024-09-26

**Authors:** Justyna Werner, Daria Mysiak

**Affiliations:** Institute of Chemistry and Technical Electrochemistry, Faculty of Chemical Technology, Poznan University of Technology, Berdychowo 4, 60-965 Poznan, Poland

**Keywords:** thin film microextraction, natural deep eutectic solvent, aspartame, acesulfame-K, parabens, benzoic acid, sorbic acid, energy drinks, isotonic drinks

## Abstract

An eco-friendly method for the determination of sweeteners (aspartame, acesulfame-K) and preservatives (benzoic acid, sorbic acid, methylparaben, ethylparaben) in functional beverages and flavoured waters using thin film microextraction (TFME) and high-performance liquid chromatography with UV detection (HPLC-UV) was proposed. A series of fourteen green and renewable solidified natural deep eutectic solvents (NADESs) were prepared and tested as ‘eutectosorbents’ in TFME for the first time. In the proposed method, the NADES containing acetylcholine chloride and 1-docosanol at a 1:3 molar ratio was finally chosen to coat a support. Four factors, i.e., the mass of the NADES, pH of the samples, extraction time, and desorption time, were tested in the central composite design to select the optimal TFME conditions. Limits of detection were equal to 0.022 µg mL^−1^ for aspartame, 0.020 µg mL^−1^ for acesulfame-K, 0.018 µg mL^−1^ for benzoic acid, 0.026 µg mL^−1^ for sorbic acid, 0.013 µg mL^−1^ for methylparaben, and 0.011 µg mL^−1^ for ethylparaben. Satisfactory extraction recoveries between 82% and 96% were achieved with RSDs lower than 6.1% (intra-day) and 7.4% (inter-day). The proposed ‘eutectosorbent’ presented good stability that enabled effective extractions for 16 cycles with recovery of at least 77%. The proposed NADES-TFME/HPLC-UV method is highly sensitive and selective. However, the use of a solid NADES as a sorbent, synthesized without by-products, without the need for purification, and with good stability on a support with the possibility of reusability increases the ecological benefit of this method. The greenness aspect of the method was evaluated using the Complex modified Green Analytical Procedure Index protocol and is equal to 84/100.

## 1. Introduction

Functional drinks, such as isotonic drinks, energy drinks, and flavoured waters are very popular, especially among young people, which is why their production is currently increasing. In addition to substances important for fulfilling a specific function, the beverages often contain flavours, colorants, artificial sweeteners, and preservatives, as evidenced by the long list of substances on the label [1,2,3]. Aspartame has not enjoyed a good reputation for years, since methanol is one of the products of its decomposition in the human body [2,4]. The most commonly used preservatives in beverages are benzoic acid with its sodium salt, and sorbic acid with its potassium salt, which may have an allergenic effect, and the cumulative content of these preservatives may cause problems with concentration and hyperactivity, especially in children [2,3]. Parabens, which are suspected to exhibit an endocrine effect, are also approved for use as food additives [5].

It is important that the content of sweeteners and preservatives in food and beverages is continuously controlled, and the liquid chromatography technique with various detectors is mainly used to determine these food additives [6,7,8,9,10,11]. Liquid phase microextraction techniques such as dispersive liquid-liquid microextraction (DLLME) [12,13,14,15,16] and ultrasound-assisted surfactant-enhanced emulsification microextraction (UASEME) [17], and solid phase (micro)extraction techniques such as solid phase microextraction (SPME) [18], thin film microextraction (TFME) [19], stir-bar sorptive extraction (SBSE) [20], and solid phase extraction (SPE) [21] are used for isolation and preconcentration of selected sweeteners and preservatives from foods, beverages, and environmental samples.

The most important solvent-free technique—solid phase microextraction (SPME)—was developed in its basic form in 1990 by Prof. J. Pawliszyn and gave rise to the development of “micro” techniques based on sorbents [22]. Due to the popularity of the SPME technique over the last 30 years, many new solutions have been proposed to increase the efficiency of extraction, including (1) new solutions in the geometry of the support (fiber, mesh, needle, stirrer, tube), (2) automation and miniaturization of SPME, (3) new sorbents, and (4) possibilities of using the SPME technique to analyze complex samples [23,24,25].

Among the many varieties of the SPME technique, special attention should be paid to thin film microextraction (TFME), which differs from classic SPME in terms of the geometry of the support, since a support with a flat and therefore more developed surface is used instead of the SPME fiber. Thus, the area of the sorbent in the form of a thin film on the support is higher. The first TFME device was proposed in 2003 [26], and since then many modifications have been made in both the shape of the media and the base materials (carbon meshes, stainless steel meshes, paper, cork) [27,28].

Deep eutectic solvents (DESs) were first introduced by Abbott et al. in 2003 [29] and are defined as eutectic mixtures that consist of at least one hydrogen bond donor (HBD) and at least one hydrogen bond acceptor (HBA), in which the formation of hydrogen bonds is the most important factor for obtaining these compounds. These compounds can be synthesized in various donor-acceptor combinations at various molar ratios. The possibilities for designing DESs are almost limitless. DESs exhibit similar properties to ionic liquids, but their synthesis is much easier and faster, without the need for purification. They are also cheaper, less toxic, non-flammable, and often biodegradable. What is important is the fact that reaching the eutectic point results in obtaining a mixture with a much lower melting point compared to the melting points of the individual HBD and HBA from which a given DES was created [30,31]. Moreover, the low eutectic point allows the synthesis of DESs that can exist as liquids at room temperature, but also as solids, which allows their use in microextraction techniques as extractants [32] and sorbents [33].

Therefore, DESs have been used for many years as effective extractants in solvent microextraction techniques, mainly in ultrasound-assisted emulsification microextraction (USAEME) and DLLME, because they are a “green” alternative to volatile, often toxic organic extractants [32]. In the case of solvent-free techniques, DESs are not yet popular (they are used mainly as modificators or additives to metal-organic frameworks, graphene, molecularly imprinted polymers, and other known sorbents), but considering their ability to solidify at room temperature, their potential for use in this technique is highly promising [33]. Our research team has developed several analytical methods based on the use of DESs as sorbents [34,35] and of hybrid materials composed of DES and poly(dimethylsiloxane) (PDMS) [36,37] for the determination of contaminants such as parabens, bisphenols, formaldehyde, and herbicides in environmental samples.

In 2011, Verpoorte et al. [38] introduced the term natural deep eutectic solvents (NADESs), which they defined as eutectic mixtures of HBD and HBA formed by compounds occurring in the metabolic pathways and cells of living organisms. In addition to the property characteristic of DESs, NADESs exhibit one significant advantage—they are completely “green” [39,40].

The aim of the present study was to develop a green, simple, and efficient NADES-TFME method for the isolation and preconcentration of aspartame (ASP), potassium acesulfame (ACE-K), benzoic acid (BA), sorbic acid (SA), methylparaben (MP), and ethylparaben (EP) in samples of functional beverages and flavoured waters, followed by HPLC-UV analysis. The structures and physicochemical properties of selected sweeteners and preservatives analyzed in this study are presented in Table S1 (Supplementary Materials).

The main novelty of the proposed method includes the synthesis of a series of fourteen NADESs that are solid at room temperature and consist of HBA (acetylcholine chloride, betaine hydrochloride, glucose), and HBD (1-eicosanol, 1-docosanol, docosanoic acid) at 1:1 and 1:3 molar ratios. Proposed ‘eutectosorbents’ used as a coating material for TFME were studied prior to the optimization of extraction factors using a response-surface design approach [41,42]. The green aspects of the proposed method were evaluated by the Complex modified Green Analytical Procedure Index (Complex MoGAPI) protocol [43].

## 2. Results and Discussion

### 2.1. Synthesis of NADESs

Focusing on green aspects, a series of green NADESs, solid at room temperature, were designed. These compounds exhibit all the features defined for NADESs, such as the connection of HBA and HBD with hydrogen bonds or the eutectic point. However, the atypical property for this particular series is their solidification and emergence as solids at room temperature. The chemical structure of NADESs and their sorption surface give them the potential to be used as sorbents, and they can even be defined as ‘eutectosorbents’. The substrates used for NADES synthesis included glucose, acetylcholine chloride, betaine hydrochloride (as HBA), long-chain carboxylic acids, and long-chain alcohols (as HBD). The selected substrates are safe for the environment and humans and/or occur in biological cycles of living organisms.

In order for the obtained NADESs to be proposed as so-called ‘eutectosorbents’ in microextraction techniques, they should meet the following criteria: (1) insolubility in water and in organic solvent(s) commonly used for desorption or as eluents; (2) liquid state at a temperature of 80–120 °C, with the possibility of their application to the support using the immersion method with uniform solidification at room temperature; and (3) stable deposition on the support in the form of a thin film even during the intensive shaking necessary at the extraction and desorption stage. Taking into account the above criteria, among the many compounds, 14 NADESs were selected for this experiment (7 each at 1:1 and 1:3 molar ratios of HBA to HBD), the structures of which are presented in Table 1.

### 2.2. Selection of ‘Eutectosorbents’ and Desorption Solvent

**Figure 1:**
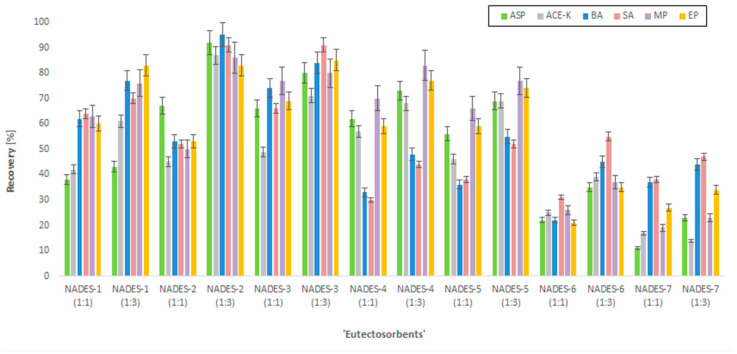
The recovery rates of the analytes (ASP, ACE-K, BA, SA, MP, EP) using selected NADESs.

Before starting the central composite design (CCD) experiment, the selection of the most efficient NADESs as the ‘eutectosorbents’ was conducted. For this purpose, 14 different NADESs were tested (Table 1). These compounds were coated on mesh supports to achieve a mass of the thin film of the NADES equal to approx. 35 mg. The supports with ‘eutectosorbents’ were immersed in bottles containing 80 mL of ultrapure water, to which 50 µg mL^−1^ of each analyte (ASP, ACE-K, BA, SA, MP, EP) and 10 mg of NaCl were added, and then the pH was adjusted to approx. 4 (acetate buffer). Afterwards, solutions were shaken for 30 min (extraction), and the supports with ‘eutectosorbents’ were transferred to test tubes containing 2 mL of acetonitrile and shaken for 20 min (desorption). Subsequently, the content of the analytes was determined by HPLC-UV, and the chromatographic peak areas were used to calculate the recovery rates. Of the tested ‘eutectosorbents’, the eutectic mixture NADES-2 (1:3) achieved the highest recovery value, which prompted its selection for further experiments (Figure 1).

As mentioned earlier, acetonitrile was selected as the desorption solvent. Initially, the solubility and stability of the support with the NADESs in acetonitrile, methanol, ethanol, and acetone were tested. In ethanol and acetone, the ‘eutectosorbents’ separated from the support, causing the solvent to become turbid. However, even a 24-h immersion of the supports with ‘eutectosorbents’ in acetonitrile or methanol and their intensive shaking did not cause the destruction or swelling of the ‘eutectosorbents’. Ultimately, acetonitrile was selected as the desorption solvent due to being less harmful than methanol.

### 2.3. Characterization of the Selected ‘Eutectosorbent’

#### 2.3.1. SEM Images

**Figure 2:**
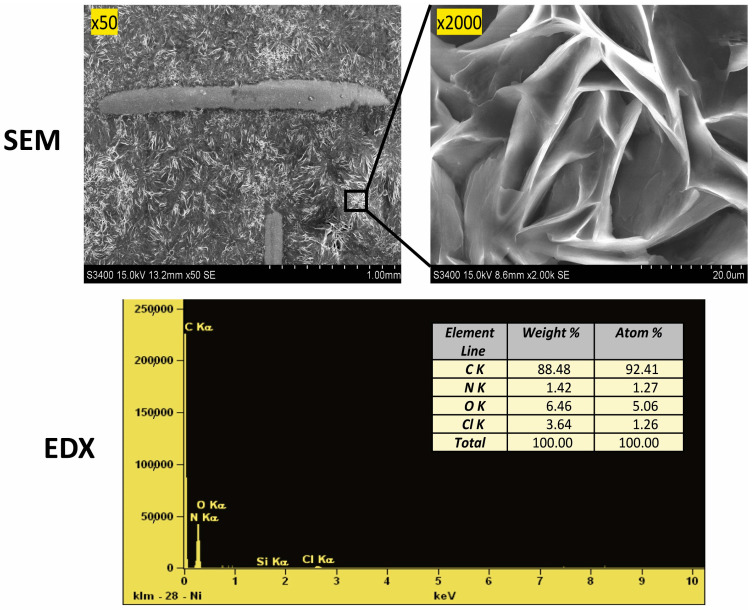
Confirmation of the structure (SEM images) and composition (EDX analysis) of the selected ‘eutectosorbent’ NADES-2 (1:3).

Scanning electron microscope (SEM) images were taken, as shown in Figure 2. The structure shown at 50× magnification confirms the precise filling of the mesh support, which affects the repeatability of their mass. The photo magnified at 2000× shows significant porosity in the form of cracks in the ‘eutectosorbent’ structure.

#### 2.3.2. Energy Dispersive X-ray Spectroscopy Analysis

The elemental composition was confirmed using energy dispersive X-ray spectroscopy (EDX). Microscopic quantitative and qualitative EDX analysis was performed on the surface of the prepared sorbent to determine the percentage composition of the sorbent (Figure 2). It was confirmed that the ‘eutectosorbent’ contains the elements carbon, oxygen, chlorine, and nitrogen, and their respective ratios match the composition of AcChCl:DcOH (1:3).

#### 2.3.3. Fourier-Transform Infrared Spectroscopy

**Figure 3:**
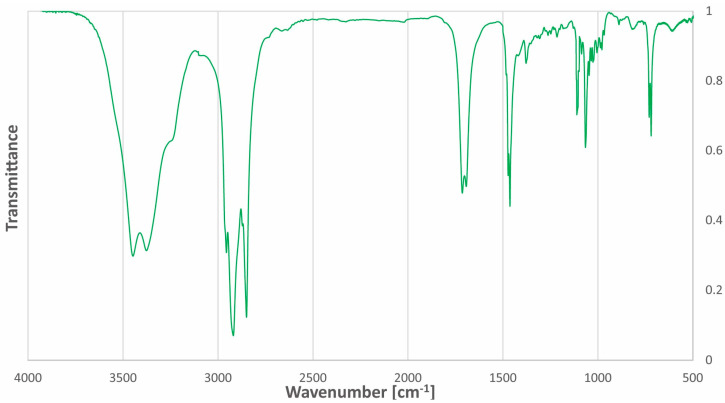
FT-IR spectrum of the ‘eutectosorbent’ acetylcholine chloride: 1-docosanol (1:3).

The Fourier-transform infrared (FT-IR) spectrum of acetylcholine chloride:1-docosanol (1:3) is mainly characterized by the presence of intermolecular hydrogen bonds in the range of 3600–3200 cm^−1^. The FT-IR spectrum of the NADES has stretching bands of C-H bonds of hydrocarbon chains (2923 cm^−1^ and 2854 cm^−1^) and stretching bands of C-H bonds of CH_3_ groups at 2955 cm^−1^. The spectrum of the NADES additionally contains a band characteristic of the C=O bond in the acetyl group (1705 cm^−1^) from the acetylcholine moiety. Two bands near 3447 cm^−1^ and 3374 cm^−1^ and characteristic of N-H bonds are visible together with the broad band of the hydrogen bond. The FT-IR spectrum of acetylcholine chloride:1-docosanol (1:3) is presented in Figure 3.

### 2.4. Response Surface Based on the Central Composite Design

The central composite design (CCD) methodology has the advantage of reducing the number of trials and more accurately finding the optimal values of the different factors affecting the extraction performance, and simultaneously detecting the presence of interactions between them. In contrast to the “one variable at the time” approach, CCD constructs experiments through statistical analysis with several factors [41,42].

In this experiment, four factors were taken into account, which seemed important for increasing the efficiency of TFME. The mass of the ‘eutectosorbent’ (in the range from 10 to 50 mg) was selected as factor A in order to check whether the mass of the thin film had an effect on the extraction efficiency. Factor B was the pH of the sample (in the range from 3 to 7). This range was selected by taking into account the pK_a_ of all analytes [44,45,46] and the pH values of the beverage samples. The amount of added salt (salt effect) was not taken into account in the CCD as a factor, because the actual samples contained a lot of mineral salts; therefore, adding additional salt was considered unnecessary. Factors C and D are the extraction time and desorption time, respectively, which were checked in the range from 5 to 25 min. Levels (−α, −1, 0, +1, +α) of selected factors are presented in Table 2.

A plan of the experiment with 29 runs that were randomly performed was generated in Statistica software (version 14.0.0.15). After completion of the runs under the given conditions, the values of the obtained response results (as peak areas) were recalculated based on the recoveries of ASP, ACE-K, BA, SA, MP, and EP (Table S2, Supplementary Materials).

**Figure 4:**
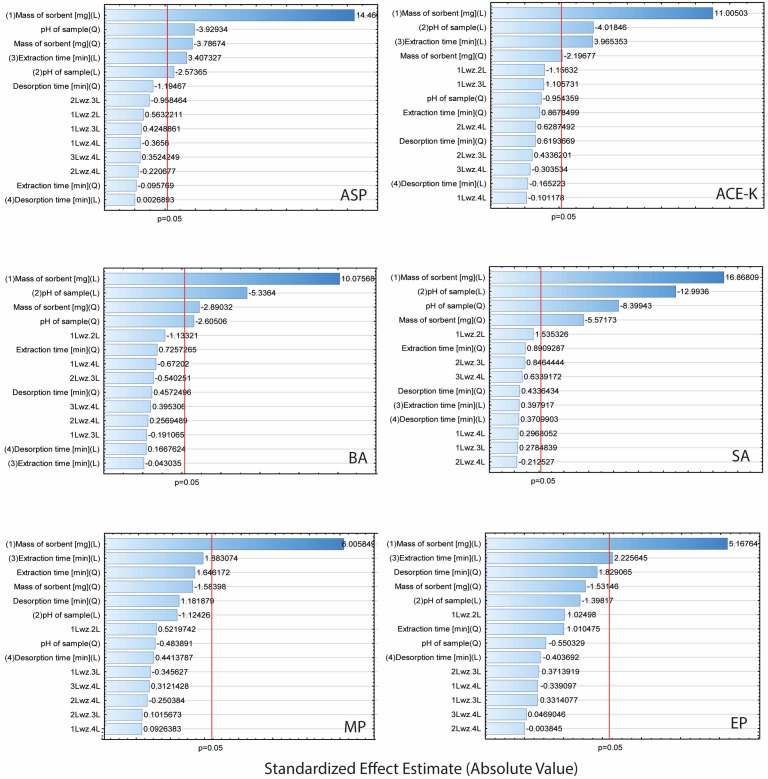
Pareto plots for extraction parameters of analytes (ASP, ACE-K, BA, SA, MP, and EP).

Taking into account the recovery values of the analytes, the experiment was analysed using Statistica software (14.0.0.15). The Pareto plots (Figure 4) illustrate the contribution of each factor (A–D) and indicate the impact of interactions on the efficiency of the TFME. Factors with a standardized effect extending beyond the red line (*p* = 0.05) were considered statistically significant.

**Figure 5:**
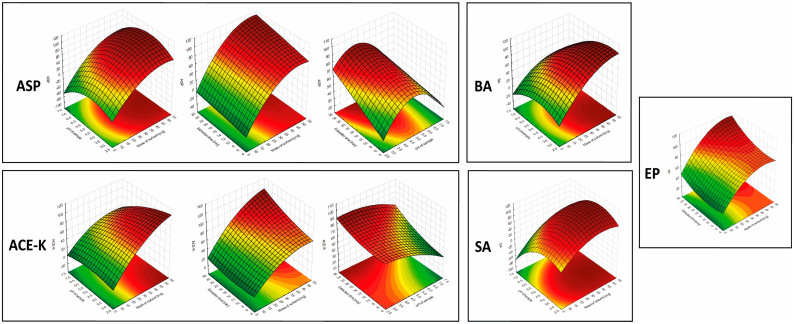
3D-graphs of correlations between significant parameters for analytes.

The results showed that the mass of the sorbent was significant in the extraction of each of the analytes (for all analytes in the linear approach, and for ASP, ACE-K, BA, and SA in the quadratic approach). Furthermore, for ASP, ACE-K, BA, and SA (compounds with pK_a_ values in the range of 3.5–4.5) the change of pH was a significant factor. The extraction time was significant for ASP, ACE-K, MP, and EP. Desorption time was insignificant for all analytes. Therefore, by taking into account the critical values and 3D-graphs of correlations between factors generated by Statistica software (version 14.0.0.15), the most beneficial conditions common to the analytes were selected. For the developed NADES-TFME/HPLC-UV method, (A) 50 mg of ‘eutectosorbent’, (B) pH = 4.5, (C) extraction time equal to 22 min, and (D) desorption time equal to 6 min were selected. 3D-graphs for statistically significant factors are presented in Figure 5, and 3D-graphs for statistically insignificant factors are presented in Figures S1–S6 in Supplementary Materials. The correlation between the observed and statistically predicted analytical signals for the experiments is shown in Figures S7–S12 in Supplementary Materials.

Analysis of variance (ANOVA) for the response surface linear and quadratic models for ASP, ACE-K, BA, SA, MP, and EP is presented in Supplementary Materials in Tables S3–S8, respectively, and the significance of each coefficient determined by F-values (variation of the mean value) and *p*-values (probability) is also given. The model turned out to be highly predictive for the experimental results.

### 2.5. Analytical Performance

Stock solutions of sweeteners (ASP, ACE-K) and preservatives (BA, SA, MP, EP) were prepared by dissolving 2 mg of each of them in 5 mL of ultrapure water. From stock solutions, standard solutions were obtained by appropriate dilution with the following concentrations: 0.05, 0.1, 0.5, 1, 5, 10, 20, 50, and 100 µg mL^−1^. Then, calibration graphs were obtained for ASP, ACE-K, SUC, BA, SA, MP, and EP. Linear regression was used to determine the linearity parameter and the determination coefficient of the optimized method. Since the determination coefficient for all calibration curves was not within the acceptable range of values, the selected calibration curve points were rejected. Thus, the range of linearity for ASP, ACE-K, BA, and SA was 0.1–100 µg mL^−1^, and for MP and EP it was 0.05–50 µg mL^−1^. The limit of detection was calculated as three times the signal-to-noise ratio. The obtained values were equal to 0.022 µg mL^−1^ for ASP, 0.020 µg mL^−1^ for ACE-K, 0.018 µg mL^−1^ for BA, 0.026 µg mL^−1^ for SA, 0.013 µg mL^−1^ for MP, and 0.011 µg mL^−1^ for EP. The limit of quantification is equal to 3.3 times the LOD value. For this experiment, the theoretical enrichment factor was expected to be 66.7, but calculated values were in range from 58 to 64 for the analyzed sweeteners and preservatives. The precision of the method expressed as the RSD (%) was determined in spiked flavoured water samples and at levels of 10 µg mL^−1^ and analyzed intra-day and inter-day. The RSD values were lower than 6.1% for the intra-day analysis and lower than 7.4% for the inter-day analysis. Validation parameters are summarized in Table 3.

### 2.6. The NADES-TFME/HPLC-UV Method on Real Samples

Method accuracy was evaluated in functional beverages (two energy drinks and two isotonic drinks) and two flavoured waters, non-spiked and spiked with 5 µg mL^−1^ or 50 µg mL^−1^ of all analytes. For each concentration level, extraction efficiency was determined by comparing the amount extracted from the sample with the amount measured in the non-spiked sample. The NADES-TFME method showed a suitable accuracy (from 84 to 97%) for all analytes at the two concentration levels, with good repeatability (RSD 3.3–8.7%). Using the NADES-TFME method, it was confirmed that the concentration of detected sweeteners and preservatives was in accordance with the information on the labels of the analysed beverages. It was also confirmed that no MP or EP were added to the analysed beverages. The results are summarized in Table 4. Retention times are 3.2 min for MP, 4.8 min for EP, 7.7 min for BA, 8.6 min for SA, 11.9 min for ACE-K, and 13.5 min for ASP. Chromatograms of (A) non-spiked isotonic drinks and (B) those spiked with 50 µg mL^−1^ of each determined analytes are presented in Figure S13 (Supplementary Materials).

### 2.7. Reusability and Reproducibility of ‘Eutectosorbent’

The reusability of AcChCl:DcOH (1:3) was evaluated after repeated extractions of analytes from the flavoured water sample. After the first TFME cycle, the thin film of ‘eutectosorbent’ was washed and conditioned with 10 mL of ultrapure water and 5 mL of 2.5% NaCl. Analytical signals for analytes were reproducible during 16 cycles (recoveries: 80–88% for ASP, 84–93% for ACE-K, 79–89% for BA, 81–90% for SA, 77–88% for MP, and 78–91% for EP). In the 17th and 18th cycles, the recoveries decreased significantly, especially for ASP (reduced to 42%), ACE-K (reduced to 48%), MP (reduced to 40%), and EP (reduced to 49%), as shown in the graph (Figure S14, Supplementary Materials). No carry-over effect was observed in subsequent cycles, because analytical signals were also checked after washing and conditioning of the ‘eutectosorbent’. According to this green approach, the developed AcChCl:DcOH (1:3) is very stable on the support and is also in contact with solvents, enabling, its repeated use.

### 2.8. Greenness Evaluation

**Figure 6:**
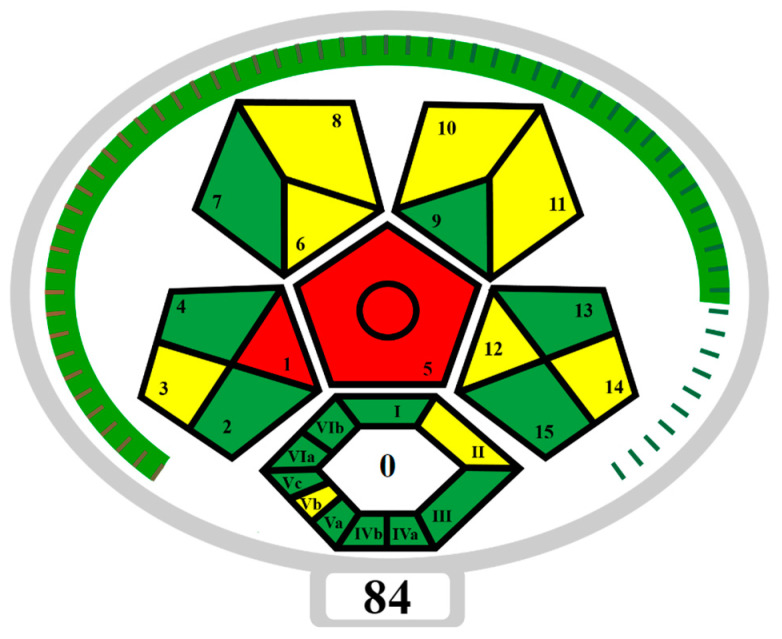
ComplexMoGAPI pictogram that presents the “green” nature of the NADES-TFME/HPLC-UV method. SAMPLE PREPARATION: 1—Collection, 2—Preservation, 3—Transport, 4—Storage, 5—Type of method, 6—Scale of extraction, 7—Solvents/reagents used, 8—Additional treatment; REAGENT AND SOLVENTS: 9—Amount, 10—Health hazard, 11—Safety hazard, 12—Energy, 13—Occupational hazard, 14—Waste, 15—Waste treatment, YIELD AND CONDITIONS: I—Yield, II—Temperature/time; RELATION TO GREEN ECONOMY: III—Number of rules met; REAGENTS AND SOLVENTS: IVa—Health hazard, IVb—Safety hazard, INSTRUMENTATION: Va—Technical setup, Vb—Energy, Vc—Occupational hazard; WORKUP AND PURIFICATION: VIa—Workup and purification of the end product, VIb—Purity; GREEN—3 points, YELLOW—2 points, RED—1 point [43].

The developed NADES-TFME/HPLC-UV analytical procedure for determining sweeteners and preservatives in functional beverages and flavoured waters was also assessed in terms of “greenness” using ComplexMoGAPI software (https://fotouhmansour.github.io/ComplexMoGAPI/, accessed on 16 September 2024) [43]. Taking into account all the important factors at the stage of ‘eutectosorbent’ synthesis, sample preparation, and conditions of analysis, the ComplexMoGAPI software generated a pictogram and greenness assessment of the proposed method equal to 84/100 (Figure 6). Based on the obtained results of the greenness assessment, the developed method of ‘eutectosorbent’ synthesis and the analytical protocol can be considered green. The main advantage is the elimination of solvents and toxic reagents from the procedure and the minimization of the use of reagents (such as acetonitrile or acetate buffer) that may affect the environment. In order to reduce the negative impact on the environment, the number of analytes used during a single analysis was increased (ASP, ACE-K, BA, SA, MP, EP), and CCD was used to select optimal TFME parameters. An important advantage is the use of a ‘eutectosorbent’ synthesized in a short time (0.5 h), obtained by a quantitative combination of HBD and HBA, without the need for purification, without by-products, and with the possibility of reusing the ‘eutectosorbent’ on the support up to 16 times. According to ComplexMoGAPI, the weak points of the developed method (red fields on the pictogram) include off-line sample collection and the requirement for extraction at the sample preparation stage.

### 2.9. Comparison of Analytical Procedures to Determine Sweeteners and Preservatives

Microextraction techniques are often used for the enrichment and determination of selected sweeteners and preservatives in food, beverage, or environmental samples. Most commonly, liquid phase microextraction techniques of different variants [13,14,15,16,17] are used, but solid phase (micro)extraction techniques are also popular [18,19,20,21] (Table 5). The proposed approach enabled the use of environmentally friendly and renewable ‘eutectosorbents’, since room-temperature solid NADESs can be prepared from natural and green compounds. The NADES-TFME/HPLC-UV method provides satisfactory analytical performance for the preconcentration and determination of sweeteners and preservatives with LOD and RSD values comparable to other analytical procedures.

## 3. Materials and Methods

### 3.1. Reagents

Ultrapure water used in the experiments was prepared by reverse osmosis in a Demiwa system from Watek (Ledec and Sazavou, Czech Republic), followed by double distillation using a Heraeus Bi 18 E quartz apparatus (Hanau, Germany). Standards of aspartame (ASP), potassium acesulfame (ACE-K), benzoic acid (BA), sorbic acid (SA), methylparaben (MP), and ethylparaben (EP) with at least 99% purity were purchased from Sigma-Aldrich (Darmstadt, Germany). Fresh stock solutions (400 µg mL^−1^ in ultrapure water) of all analytes were prepared every week and stored in a freezer at −4 °C. Acetate buffer was used to adjust the pH of the samples and to prepare the chromatographic eluent phase. Acetonitrile (MeCN, gradient grade for LC, LiChroSolv^®^) was purchased from Merck (Darmstadt, Germany) and used during the desorption of analytes and to prepare the chromatographic eluent phase. Docosanoic acid (DCA, purity 99%), 1-docosanol (DcOH, purity 99%), and 1-eicosanol (EiOH, purity 98.5%) were purchased from Merck (Darmstadt, Germany) and used as hydrogen bond donors in NADES synthesis. Acetylcholine chloride (AcChCl, purity ≥ 99%), betaine hydrochloride (BeCl, purity ≥ 98%), and glucose (GLU, purity 98%) were purchased from Merck (Darmstadt, Germany) and used as hydrogen bond acceptors in NADES synthesis.

### 3.2. Real Samples

Samples of isotonic drinks, energy drinks, and flavoured waters were bought in local shops (Poznań, Poland). Samples were opened and degassed (10 min in an ultrasound bath) directly prior to the analytical procedure.

### 3.3. Apparatus and Lab Equipment

A glass fibre mesh used as a support for the thin film of ‘eutectosorbent’ was purchased from Golden Plast (Poland). A magnetic stirrer with heating up to 550 °C and stirring up to 1500 rpm (SunLab SU1300, Mannheim, Germany) was used for synthesis of NADESs. A pH meter (EL20, Mettler Toledo, Switzerland) with a lab pH electrode (type LE407) was used for pH measurements. An orbital shaker (PSU-20 i, Grant-Bio, Royston, UK) with a platform for shaking bottles with a capacity of up to 100 mL was used for extraction. A Hewlett Packard 1100 HPLC system with a UV detector was used for the determination of ASP, ACE-K, BA, SA, MP, and EP. The analytes were separated in a reversed-phase mode using a LiChrosorb^®^ RP-18 HPLC column (250 mm × 4.6 mm I.D., 5 μm particle size) purchased from Merck Millipore (Darmstadt, Germany). ChemStation software (version Rev.A.06.01) was used to acquire and process the chromatographic data. The Fourier-transform infrared spectroscopy (FT-IR) spectra of the ‘eutectosorbents’ and substrates were obtained using the IFS 66 v/S FT-IR spectrometer from Bruker Optic (Ettlingen, Germany). The scans (from 500 to 4000 cm^−1^) were performed in reflection mode using the attenuated total reflection (ATR) module from Perkin Elmer (Waltham, MA, USA). A scanning electron microscope (SEM, S-3400 N, Hitachi, Tokyo, Japan) was used to study the surfaces and morphologies of the thin films of the ‘eutectosorbent’ on the supports. The Cressington Carbon Coater was used to coat the films with conductive carbon material for better image quality. Thermo Scientific NSS spectral imaging software was used for elemental analysis of ‘eutectosorbents’ using the energy dispersive X-ray (EDX) spectrometry technique.

### 3.4. Preparation of NADESs

NADESs used in the experiment were synthesized by mixing selected HBAs (acetylcholine chloride, betaine hydrochloride or glucose) and HBDs (docosanoic acid, 1-docosanol or 1-eicosanol) at 1:1 and 1:3 molar ratios. Substrates were weighed and transferred to 20 mL glass vials. Each mixture was stirred and heated to dissolve the substrates and for another 30 min to achieve the eutectic point and obtain homogenous NADESs. Then, the NADESs were cooled off to room temperature to solidify. Structures of NADESs that were synthesized and used in this experiment are shown in Table 1**.**

### 3.5. Experimental Design

The extraction efficiency depends on several factors; therefore, multivariate methods of optimization, including factorial designs and response surface methodology (RSM) [41,42], have been used to evaluate the main and interactive effects of several variables simultaneously with a reduced number of experimental runs. The NADES-TFME conditions were optimized using a central composite design (CCD), and factors selected for optimization included (A) the mass of the sorbent, NADES [mg]; (B) the pH of the sample; (C) the extraction time [min]; and (D) the desorption time [min]. The factor levels are shown in Table 2. Twenty-nine experiments were generated in Statistica software 14.0.0.15 and were executed in a random order to ensure the independence of the results by minimizing the effect of uncontrolled factors. All statistical analysis, i.e., generation of response surfaces, Pareto plots (the significance level was set at *p* < 0.05), 3D-graphs for significant and insignificant factors, critical values of factors (A–D), and predicted/observed values, were accomplished using the Statistica 14.0.0.15 (TIBCO Software Inc., Santa Clara, CA, USA).

### 3.6. NADES-TFME Procedure

A strip mesh (made from glass fibre) was used as a support for thin films of AcChCl:DcOH (1:3) as a ‘eutectosorbent’. The strips of supports were immersed in a heated NADES at the same depth. On each support mesh, the coatings were distributed evenly. For better dipping process control, the mesh was weighed before and after the coating. The mass of the NADES covering the support ranged from 49.8 to 50.4 mg and filled the mesh entirely. The film thickness was equal to 300 µm. To preconcentrate the analytes using the TFME technique, 80 mL of the degassed beverage samples were placed in glass bottles with a capacity of 100 mL, and acetate buffer was added to adjust the pH to 4.5. Supports with thin films of AcChCl:DcOH (1:3) as a ‘eutectosorbent’ were hooked into the PTFE plug so that total immersion of the thin films in the solution of the samples was achieved. All samples were transferred to an orbital shaker with a platform for bottles. The shaking process was performed at 230 rpm and lasted for 22 min. After extraction, supports with ‘eutectosorbent’ coatings were moved to test tubes, and 1.2 mL of acetonitrile was added to each tube. For the desorption step, all test tubes were placed on a shaker set on 230 rpm for 6 min. After desorption, samples were analysed with the HPLC-UV technique.

### 3.7. Chromatographic Conditions

The HPLC analysis was conducted in a reversed-phase system. The flow rate of the mobile phase was equal to 1.0 mL min^−1^. Isocratic elution was performed using a mixture of acetate buffer (pH = 4.5) and acetonitrile at a 75/25 (*v*/*v*) ratio as the mobile phase. The absorption was selected based on scanning in the range from 210 to 260 nm and finally measured at a wavelength of λ = 240 nm.

## 4. Conclusions

In this study, an innovative and completely ecological series of NADESs, solid at room temperature, was proposed. These compounds were designed and synthesized without the need for their purification, without obtaining by-products, in about 30 min. By definition, these compounds are deep eutectic solvents which solidify at temperatures below their eutectic point, thus allowing their use as sorbents and their designation as ‘eutectosorbents’. Their use in solvent-free microextraction techniques, insolubility in water and organic solvents, stability on a support (in this case, a mesh support from glass fibre was used), and the possibility of their multiple uses are important. In this study, among the synthetized ‘eutectosorbents’ proposed, one of them, acetylcholine chloride:1-docosanol (in the molar ratio 1:3) was identified for its efficient extraction of popular sweeteners and preservatives in functional beverages and flavoured water samples. Moreover, the structures of these compounds and their physicochemical and sorption properties allow for the continuation of research in this area and testing of their properties with the use of other analytes. In addition, a future trend could be the preparation of hybrid materials from ‘eutectosorbents’, biopolymers, and biowaste, which could be characterized by higher sorption capacities and eco-friendliness. The limitation of the use of ‘eutectosorbents’ is their low melting point (80–120 °C), which does not allow thermal desorption, and therefore they cannot be used in systems with gas chromatography.

## Figures and Tables

**Table 1:** Structures of NADESs used as ‘eutectosorbents’ in TFME.

Symbol of NADES	Structure of HBA	Structure of HBD	HBA/HBD (Molar Ratio)	Solidification Point
NADES-1	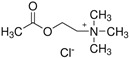 acetylcholine chloride (AcChCl)	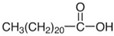 docosanoic acid (DCA)	1:1 1:3	102 °C 95 °C
NADES-2	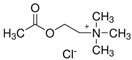 acetylcholine chloride (AcChCl)	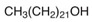 1-docosanol (DcOH)	1:1 1:3	111 °C 103 °C
NADES-3	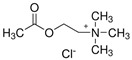 acetylcholine chloride (AcChCl)	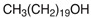 1-eicosanol (EiOH)	1:1 1:3	108 °C 101 °C
NADES-4	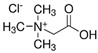 betaine chloride (BeCl)	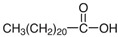 docosanoic acid (DCA)	1:1 1:3	122 °C 109 °C
NADES-5	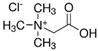 betaine chloride (BeCl)	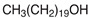 1-eicosanol (EiOH)	1:1 1:3	105 °C 97 °C
NADES-6	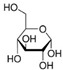 glucose (GLU)	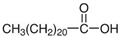 docosanoic acid (DCA)	1:1 1:3	93 °C 89 °C
NADES-7	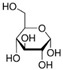 glucose (GLU)	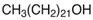 1-docosanol (DcOH)	1:1 1:3	90 °C 86 °C

**Table 2:** The experimental levels of the factors used in the central composite design.

Factor	Symbol	Levels of Factors				
		Low Axial (−α)	Low Factorial (−1)	Center (0)	High Factorial (+1)	High Axial (+α)
Mass of sorbent [mg]	A	10	20	30	40	50
pH of sample	B	3	4	5	6	7
Extraction time [min]	C	5	10	15	20	25
Desorption time [min]	D	5	10	15	20	25

**Table 3:** Validation parameters, recovery, and enrichment factor of the proposed NADES-TFME/HPLC-UV method.

Parameter		ASP	ACE-K	BA	SA	MP	EP
Linear range [µg mL^−1^]		0.1–100	0.1–100	0.1–100	0.1–100	0.05–50	0.05–50
Determination coefficient (R^2^)		0.9990	0.9993	0.9992	0.9991	0.9994	0.9995
LOD [µg mL^−1^]		0.022	0.020	0.018	0.026	0.013	0.011
LOQ [µg mL^−1^]		0.073	0.066	0.059	0.086	0.043	0.036
Precision RSD [%] (n = 4)	intra-day	3.8	4.2	5.9	5.7	3.9	6.1
	inter-day	5.7	6.6	7.4	6.9	7.2	6.5
Extraction recovery [%]		85	82	89	93	92	96
Enrichment factor		59	58	62	64	60	62

**Table 4:** Amounts of selected sweeteners and preservatives in beverages initially and with added analytes (5 and 50 µg mL^−1^).

Sample	Analytes	Declared on the Label	Initial, µg mL^−1^	Added 5 µg mL^−1^			Added 50 µg mL^−1^		
				Measured	RSD [%] (n = 4)	Recovery [%]	Measured	RSD [%] (n = 4)	Recovery [%]
isotonic drink type “light”	ASP	YES	127.41 ± 5.15	131.62 ± 6.37	4.8	84	175.74 ± 7.08	4.0	97
	ACE-K	YES	229.86 ± 9.06	234.53 ± 10.28	4.4	93	275.31 ± 9.73	3.5	91
	BA	No	nd	4.69 ± 0.39	8.3	94	46.44 ± 3.01	6.5	93
	SA	No	nd	4.55 ± 0.32	7.0	91	44.15 ± 3.26	7.4	88
	MP	No	nd	4.50 ± 0.34	7.6	90	44.12 ± 3.83	8.7	88
	EP	No	nd	4.58 ± 0.40	8.7	92	42.71 ± 4.17	7.4	86
isotonic drink with multi-fruit flavour	ASP	YES	50.44 ± 3.87	54.60 ± 4.21	7.7	86	95.39 ± 5.52	5.8	90
	ACE-K	YES	77.86 ± 3.74	82.45 ± 4.06	4.9	92	121.61 ± 5.58	4.6	88
	BA	YES	131.22 ± 7.01	135.74 ± 7.74	5.7	90	177.44 ± 8.08	4.5	92
	SA	No	nd	4.45 ± 0.38	8.5	89	46.31 ± 2.73	5.9	93
	MP	No	nd	4.69 ± 0.37	7.9	94	44.82 ± 3.87	8.6	90
	EP	No	nd	4.30 ± 0.32	7.4	86	43.19 ± 3.61	8.4	87
energy drink with vitamins	ASP	YES	167.09 ± 3.55	171.61 ± 6.26	3.7	90	211.98 ± 7.84	3.7	90
	ACE-K	YES	202.96 ± 7.29	207.29 ± 8.69	4.2	87	249.34 ± 8.26	3.3	93
	BA	No	nd	4.61 ± 0.39	8.5	92	46.24 ± 3.01	6.7	93
	SA	YES	119.41 ± 5.52	123.65 ± 7.33	5.9	85	164.98 ± 9.73	5.9	91
	MP	No	nd	4.59 ± 0.34	7.4	92	47.77 ± 2.57	5.4	96
	EP	No	nd	4.56 ± 0.26	5.7	91	44.71 ± 3.21	7.2	89
energy drink with ginseng	ASP	No	nd	4.45 ± 0.34	7.6	89	45.79 ± 2.82	6.1	92
	ACE-K	YES	250.13 ± 8.07	254.66 ± 9.35	3.7	91	295.53 ± 10.07	3.4	91
	BA	YES	128.95 ± 6.90	133.57 ± 7.27	5.4	92	174.31 ± 7.73	4.4	90
	SA	No	nd	4.71 ± 0.32	6.8	94	43.77 ± 3.84	8.7	88
	MP	No	nd	4.58 ± 0.39	8.5	92	42.86 ± 2.97	6.9	86
	EP	No	nd	4.57 ± 0.33	7.2	92	44.74 ± 3.58	8.0	90
lemon flavoured water, type “light”	ASP	Yes	80.77 ± 4.98	84.99 ± 5.88	6.9	84	125.92 ± 8.33	6.6	90
	ACE-K	No	nd	4.70 ± 0.37	7.9	94	46.56 ± 4.01	8.6	93
	BA	No	nd	4.64 ± 0.33	7.1	93	45.44 ± 3.28	7.2	91
	SA	No	nd	4.35 ± 0.38	8.6	87	47.31 ± 2.73	5.8	95
	MP	No	nd	4.37 ± 0.30	6.9	87	44.09 ± 3.01	6.8	88
	EP	No	nd	4.32 ± 0.24	5.5	86	45.69 ± 3.86	8.5	91
lemon flavoured water	ASP	No	nd	4.82 ± 0.38	7.9	96	43.42 ± 2.33	5.4	87
	ACEK	No	nd	4.43 ± 0.26	5.8	89	46.56 ± 3.41	7.3	93
	BA	Yes	95.71 ± 7.08	100.08 ± 7.37	7.4	87	140.44 ± 7.04	5.0	89
	SA	No	nd	4.66 ± 0.22	4.7	93	44.35 ± 3.43	7.7	89
	MP	No	nd	4.39 ± 0.31	7.1	88	43.68 ± 2.94	6.7	87
	EP	No	nd	4.26 ± 0.35	8.2	85	45.55 ± 3.17	7.0	91

**Table 5:** Comparison of methods dedicated to preconcentration and determination of selected sweeteners and preservatives.

Analytes	Samples	Extraction Medium	Extraction Technique	Detection	LOD [µg⋅L^−1^]	RSD [%]	Ref.
BA, SA, MP, EP, PP, BP	food	acetone/ TCM	DLLME	GC-MS	0.15–0.20 *	<5.0	[13]
BA, SA, MP, EP, PP, BP	beverages	1-decanol	DLLME-SFO	HPLC-DAD	0.02–0.05	<5.0	[14]
SA, BA	beverages, soy sauces	menthol	AA-DLLME-SFOD	HPLC-UV	0.02–0.03 **	4.0–8.0 3.0–6.0	[15]
MP, EP, PP, iPP, iBP	beverages	TBACl:OCA	LLME- SFOD	HPLC-UV	0.15–0.20 **	<6.88	[16]
BA, MP, EP, PP, BP	waters, beverages, personal care products	1-octanol	UASEME	HPLC-UV	0.3–10	<7.0	[17]
SA, BA	beverages	PDMS/DVB	HS-SPME	GC-FID	5.8–11.4	<8.6	[18]
ACE-K, SAC, CYC, ASP, SUC, NHDC	environmental waters	HLB-WAX	TF-SPME	UPLC-MS/MS	0.004–0.05	<18.3	[19]
BA, SA, EP, PP, iPP, BP, iBP	beverages, vinegar, quasi-drug drinks	PDMS	SBSE	TD-GC/MS	0.015–3.3 **	0.86–6.0	[20]
ACE-K, SAC, CYC, ASP, SUC, NHDC	environmental waters	Oasis HLB	SPE	LC-MS/MS	-	<10.0	[21]
ACE-K, ASP, BA, SA, MP, PP	functional beverages, flavoured waters	NADES (AcChCl:DcOH) (1:3)	TFME	HPLC-UV	0.01–0.03 **	<7.4	this study

* mg kg^−1^, ** µg mL^−1^; BA—benzoic acid, SA—sorbic acid, MP—methylparaben, EP—ethylparaben, PP—propylparaben, BP—butylparaben, TCM—trichloromethane, DLLME—dispersive liquid-liquid microextraction, DES—deep eutectic solvent, SFOD—solidification of floating organic droplet, AA-DLLME-OPS—air-assisted dispersive liquid-liquid microextraction procedure with organic phase solidification, iPP—isopropylparaben, iBP—isobutylparaben, TBACl—tetrabutylammonium bromide, OCA—octanoic acid, LLME-OPS—liquid–liquid microextraction based on solidification of floating organic droplet, UASEME—ultrasound-assisted surfactant-enhanced emulsification microextraction, PDMS–DVB—polydimethylsiloxane–divinylbenzene, HS-SPME—headspace solid phase microextraction, ACEK—acesulfame potassium, SAC—saccharin, CYC—sodium cyclamate, ASP—aspartame, SUC—sucralose, NHDC—neohesperidin dihydrochalcone, HLB-WAX—hydrophilic-lipophilic balanced-weak anion exchange particles, TF-SPME—thin film solid phase microextraction, PDMS—polydimethylsiloxane, SBSE—stir-bar sorptive extraction, TD-GC–MS—thermal desorption gas chromatography-mass spectrometry, SPE—solid phase extraction, AcChCl—acetylcholine chloride, DcOH—docosanol.

## Data Availability

Dataset available on request from the authors.

## Supplementary Materials

The following supporting information can be downloaded at: https://www.mdpi.com/article/10.3390/molecules29194573/s1, Table S1—Structures and properties of sweeteners and preservatives determined in this study. Table S2—The values of factors (A-D) generated by the central composite design experiments and the responses (as recovery) for analytes (ASP, ACE-K, BA, SA, MP, and EP). Table S3—Analysis of variance (ANOVA) of response surface linear and quadratic models for aspartame. Table S4—Analysis of variance (ANOVA) of response surface linear and quadratic models for potassium acesulfame. Table S5—Analysis of variance (ANOVA) of response surface linear and quadratic models for benzoic acid. Table S6—Analysis of variance (ANOVA) of response surface linear and quadratic models for sorbic acid. Table S7—Analysis of variance (ANOVA) of response surface linear and quadratic models for methylparaben. Table S8—Analysis of variance (ANOVA) of response surface linear and quadratic models for ethylparaben. Figure S1—3D-graphs of correlations between insignificant parameters for ASP: (1) AxD, (2) BxD, and (3) CxD. Figure S2—3D-graphs of correlations between insignificant parameters for ACE-K: (1) AxD, (2) BxD, and (3) CxD. Figure S3—3D-graphs of correlations between insignificant parameters for BA: (1) AxC, (2) AxD, (3) BxC, (4) BxD, and (5) CxD. Figure S4—3D-graphs of correlations between insignificant parameters for SA: (1) AxC, (2) AxD, (3) BxC, (4) BxD, and (5) CxD. Figure S5—3D-graphs of correlations between insignificant parameters for MP: (1) AxB, (2) AxC, (3) AxD, (4) BxC, (5) BxD, and (6) CxD. Figure S6—3D-graphs of correlations between insignificant parameters for EP: (1) AxB, (2) AxD, (3) BxC, (4) BxD, and (5) CxD. Figure S7—Correlation between observed and predicted values for ASP. Figure S8—Correlation between observed and predicted values for ACE-K. Figure S9—Correlation between observed and predicted values for BA. Figure S10—Correlation between observed and predicted values for SA. Figure S11—Correlation between observed and predicted values for MP. Figure S12—Correlation between observed and predicted values for EP. Figure S13—Chromatograms of isotonic drink type ‘light’: (A) non-spiked and (B) spiked with 50 µg mL^−1^ of each analyte. Figure S14—The chart of the reusability of AcChCl:DcOH (1:3) for the extraction of ASP, ACE-K, BA, SA, MP, and EP.
