# Supplementary material for: Development of Thin Film Microextraction with Natural Deep Eutectic Solvents as ‘Eutectosorbents’ for Preconcentration of Popular Sweeteners and Preservatives from Functional Beverages and Flavoured Waters
Source: Molecules. 2024 Sep 26;29(19):4573. doi: 10.3390/molecules29194573 (PMC11477994; doi:10.3390/molecules29194573)
Supplement: Supplementary file 1 [file molecules-29-04573-s001.zip › molecules-3210113-supplementary.pdf]

Supplementary Materials

Development of thin film microextraction with natural deep eutectic solvents as 'eutectosorbent' for preconcentration of popular sweeteners and preservatives from functional beverages and flavoured waters

Justyna Werner*. Daria Mysiak

Institute of Chemistry and Technical Electrochemistry. Faculty of Chemical Technology. Poznan University of Technology. Berdychowo 4. 60-965 Poznan. Poland

Table S1: Structures and properties of sweeteners and preservatives determined in this study.

**Analyte (Abbr.)**	**Symbol E**	**Structure**	**ADI [mg/kg bw/day]**	**pK_a**	**logP**
Aspartame **(ASP)**	E951	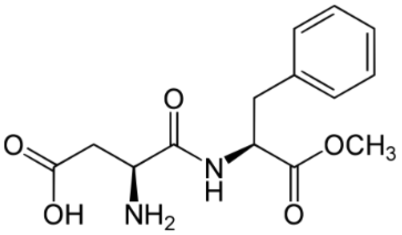	40	3.53[19]	-2.22 [19]
Acesulfame-K **(ACE-K)**	E950	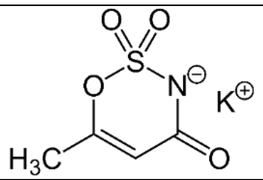	15	3.02[19]	-0.69[19]
Benzoic acid **(BA)**	E210	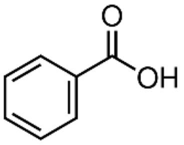	20	4.19[44]	1.87[44]
Sorbic acid **(SA)**	E200	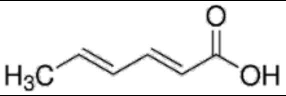	25	4.76[45]	1.33[45]
Methylparaben **(MP)**	E218	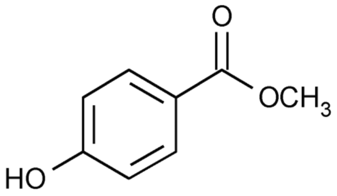	10	8.47[46]	1.91[46]
Ethylparaben **(EP)**	E214	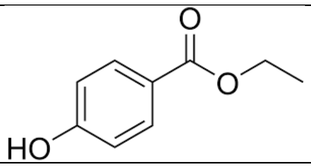	10	8.50[46]	2.34[46]

Table S2: The values of factors (A-D) generated by the central composite design experiments and the responses (as recovery) for analytes (ASP, ACE-K, BA, SA, MP and EP).

No.	Mass of sorbent [mg]	pH of sample	Extraction time [min]	Desorption time [min]	ASP	ACE-K	BA	SA	MP	EP
1	20	4	10	10	35.2	51.8	61.8	72.9	71.2	79.5
2	20	4	10	20	31.7	52.5	62.5	76.4	76.8	77.2
3	20	4	20	10	44.3	66.1	66.1	70.6	81.5	82.4
4	20	4	20	20	45.5	60.4	70.4	70.3	87.8	85.2
5	20	6	10	10	23.8	44.2	54.2	41.1	66.3	61.5
6	20	6	10	20	28.2	45.3	55.3	40.0	63.6	67.1
7	20	6	20	10	34.1	55.4	45.4	46.3	76.9	77.6
8	20	6	20	20	37.1	57.4	57.4	44.3	79.4	74.6
9	40	4	10	10	75.9	79.8	96.8	97.4	91.9	88.3
10	40	4	10	20	73.5	77.7	93.7	93.8	93.9	84.0
11	40	4	20	10	91.0	97.5	93.5	95.2	98.4	97.7
12	40	4	20	20	98.6	92.6	95.6	100.3	103.8	97.3
13	40	6	10	10	79.1	60.3	72.3	74.4	90.2	87.6
14	40	6	10	20	74.8	61.5	75.5	72.6	92.0	83.3
15	40	6	20	10	87.8	82.1	76.7	71.6	95.8	99.9
16	40	6	20	20	80.9	83.2	72.2	77.4	102.4	97.3
17	10	5	15	15	20.7	11.4	11.4	25.2	14.5	19.1
18	50	5	15	15	98.7	97.1	97.1	102.7	96.9	91.1
19	30	3	15	15	70.3	72.8	72.7	74.0	71.2	71.4
20	30	7	15	15	47.4	49.2	39.2	33.7	59.6	55.5
21	30	5	5	15	74.3	67.7	77.5	86.6	78.3	68.0
22	30	5	25	15	89.1	74.1	74.1	87.4	89.9	85.4
23	30	5	15	5	74.9	69.3	76.6	84.7	80.8	86.9
24	30	5	15	25	75.4	69.8	71.8	86.1	79.3	80.5
25 (C)	30	5	15	15	73.7	67.2	77.8	82.7	79.6	76.4
26 (C)	30	5	15	15	77.2	68.9	78.4	83.7	79.2	75.1
27 (C)	30	5	15	15	76.5	66.9	76.3	80.6	75.7	80.9
28 (C)	30	5	15	15	75.7	69.3	70.3	85.3	81.8	78.6
29 (C)	30	5	15	15	74.9	68.0	77.2	83.6	80.0	76.5

Table S3: Analysis of variance (ANOVA) for response surface linear and quadratic model for aspartame.

Source	Sum of squares	Degrees of freedom	Mean square	F-value	P-value probe >P
A	12046.72	1	12046.72	209.1002	0.000000
A^2	826.12	1	826.12	14.3394	0.002003
B	381.60	1	381.60	6.6237	0.022080
B^2	889.52	1	889.52	15.4397	0.001512
C	668.87	1	668.87	11.6099	0.004250
C^2	0.53	1	0.53	0.0092	0.925061
D	0.00	1	0.00	0.0000	0.997892
D^2	82.23	1	82.23	1.4272	0.252063
AB	18.28	1	18.28	0.3172	0.582193
AC	10.40	1	10.40	0.1805	0.677377
AD	7.70	1	7.70	0.1337	0.720129
BC	52.93	1	52.93	0.9187	0.354090
BD	2.81	1	2.81	0.0487	0.828530
CD	7.16	1	7.16	0.1242	0.729769
Error	806.57	14	57.61		
Total SS	15525.42	28			

Table S4: Analysis of variance (ANOVA) for response surface linear and quadratic model for potassium acesulfame.

Source	Sum of squares	Degrees of freedom	Mean square	F-value	P-value probe >P
A	5797.042	1	5797.042	121.1108	0.000000
A^2	230.991	1	230.991	4.8258	0.045374
B	772.935	1	772.935	16.1480	0.001269
B^2	43.596	1	43.596	0.9108	0.356091
C	752.640	1	752.640	15.7240	0.001409
C^2	36.051	1	36.051	0.7532	0.400108
D	1.307	1	1.307	0.0273	0.871130
D^2	18.362	1	18.362	0.3836	0.545623
AB	64.000	1	64.000	1.3371	0.266902
AC	58.522	1	58.522	1.2226	0.287483
AD	0.490	1	0.490	0.0102	0.920844
BC	9.000	1	9.000	0.1880	0.671171
BD	18.923	1	18.923	0.3953	0.539637
CD	4.410	1	4.410	0.0921	0.765947
Error	670.119	14	47.866		
Total SS	8535.427	28			

Table S5: Analysis of variance (ANOVA) for response surface linear and quadratic model for benzoic acid.

Source	Sum of squares	Degrees of freedom	Mean square	F-value	P-value probe >P
**A**	5846.882	1	5846.882	101.5193	0.000000
**A^2**	481.136	1	481.136	8.3540	0.011867
**B**	1640.107	1	1640.107	28.4771	0.000105
**B^2**	390.852	1	390.852	6.7864	0.020772
**C**	0.107	1	0.107	0.0019	0.966281
**C^2**	30.333	1	30.333	0.5267	0.479963
**D**	1.602	1	1.602	0.0278	0.869942
**D^2**	12.042	1	12.042	0.2091	0.654507
**AB**	73.960	1	73.960	1.2842	0.276160
**AC**	2.103	1	2.103	0.0365	0.851218
**AD**	26.010	1	26.010	0.4516	0.512509
**BC**	16.810	1	16.810	0.2919	0.597514
**BD**	3.802	1	3.802	0.0660	0.800956
**CD**	9.000	1	9.000	0.1563	0.698575
**Error**	806.313	14	57.594		
**Total SS**	9394.981	28			

Table S6: Analysis of variance (ANOVA) for response surface linear and quadratic model for sorbic acid.

Source	Sum of squares	Degrees of freedom	Mean square	F-value	P-value probe >P
**A**	5886.49	1	5886.490	284.5324	0.000000
**A^2**	642.25	1	642.250	31.0441	0.000069
**B**	3492.90	1	3492.898	168.8345	0.000000
**B^2**	1459.57	1	1459.568	70.5504	0.000001
**C**	3.28	1	3.276	0.1583	0.696693
**C^2**	16.42	1	16.421	0.7938	0.388023
**D**	2.85	1	2.847	0.1376	0.716198
**D^2**	3.89	1	3.890	0.1880	0.671155
**AB**	48.77	1	48.767	2.3572	0.146990
**AC**	1.60	1	1.604	0.0776	0.784712
**AD**	1.82	1	1.823	0.0881	0.770973
**BC**	14.82	1	14.822	0.7165	0.411540
**BD**	0.93	1	0.934	0.0452	0.834759
**CD**	8.31	1	8.314	0.4019	0.536355
**Error**	289.64	14	20.688		
**Total SS**	11876.19	28			

Table S7: Analysis of variance (ANOVA) for response surface linear and quadratic model for methylparaben.

Source	Sum of squares	Degrees of freedom	Mean square	F-value	P-value probe >P
**A**	4524.218	1	4524.218	36.07022	0.000032
**A^2**	314.699	1	314.699	2.50900	0.135520
**B**	158.535	1	158.535	1.26395	0.279812
**B^2**	29.369	1	29.369	0.23415	0.635944
**C**	444.764	1	444.764	3.54597	0.080635
**C^2**	339.895	1	339.895	2.70988	0.121984
**D**	24.435	1	24.435	0.19482	0.665679
**D^2**	175.203	1	175.203	1.39684	0.256940
**AB**	34.174	1	34.174	0.27246	0.609848
**AC**	14.983	1	14.983	0.11946	0.734762
**AD**	1.076	1	1.076	0.00858	0.927503
**BC**	1.294	1	1.294	0.01032	0.920540
**BD**	7.863	1	7.863	0.06269	0.805927
**CD**	12.221	1	12.221	0.09743	0.759533
**Error**	1755.993	14	125.428		
**Total SS**	8028.405	28			

Table S8: Analysis of variance (ANOVA) for response surface linear and quadratic model for ethylparaben.

Source	Sum of squares	Degrees of freedom	Mean square	F-value	P-value probe >P
**A**	3136.544	1	3136.544	26.70451	0.000143
**A^2**	275.473	1	275.473	2.34537	0.147936
**B**	229.608	1	229.608	1.95488	0.183820
**B^2**	35.572	1	35.572	0.30286	0.590767
**C**	581.807	1	581.807	4.95350	0.042980
**C^2**	119.927	1	119.927	1.02106	0.329414
**D**	19.141	1	19.141	0.16297	0.692537
**D^2**	392.939	1	392.939	3.34548	0.088767
**AB**	123.395	1	123.395	1.05058	0.322758
**AC**	12.900	1	12.900	0.10983	0.745245
**AD**	13.506	1	13.506	0.11499	0.739569
**BC**	16.201	1	16.201	0.13793	0.715906
**BD**	0.002	1	0.002	0.00001	0.996987
**CD**	0.258	1	0.258	0.00220	0.963252
**Error**	1644.352	14	117.454		
**Total SS**	6783.833	28			

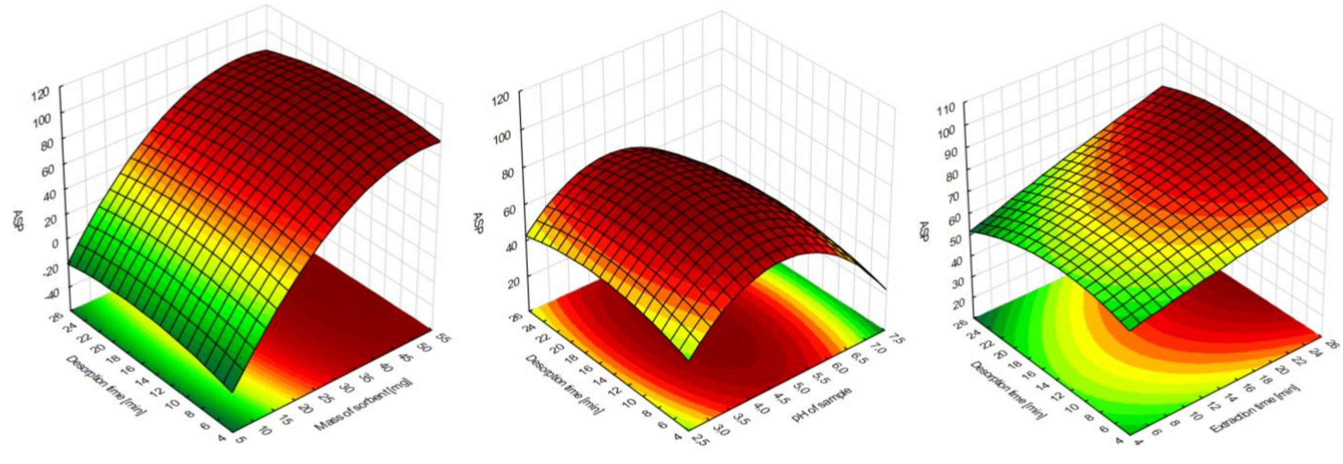

Fig. S1: 3D-graphs of correlation between insignificant parameters for ASP: (1) AxD, (2) BxD, (3) CxD.

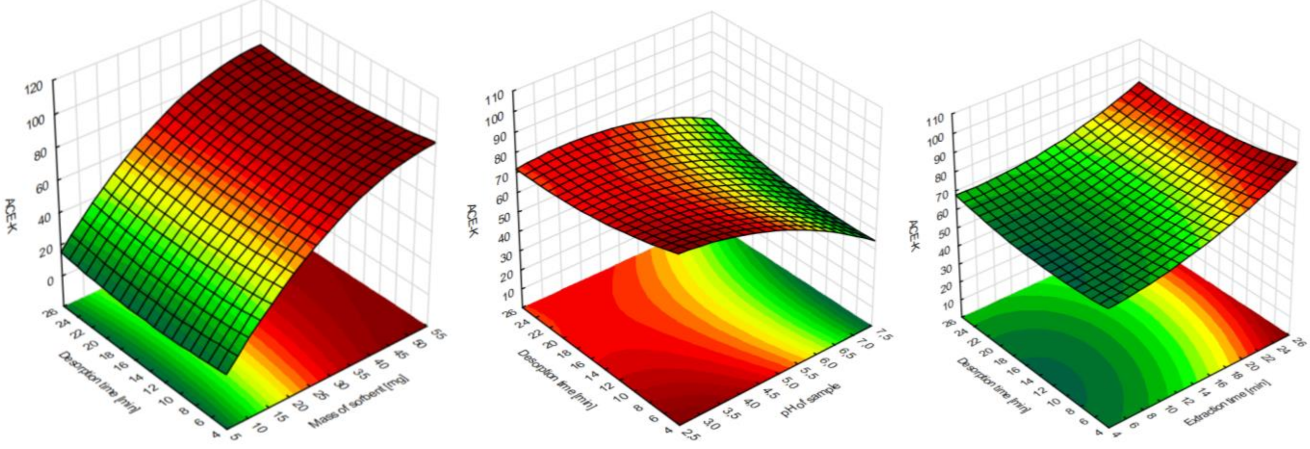

Fig. S2: 3D-graphs of correlation between insignificant parameters for ACE-K:

(1) AxD, (2) BxD, (3) CxD.

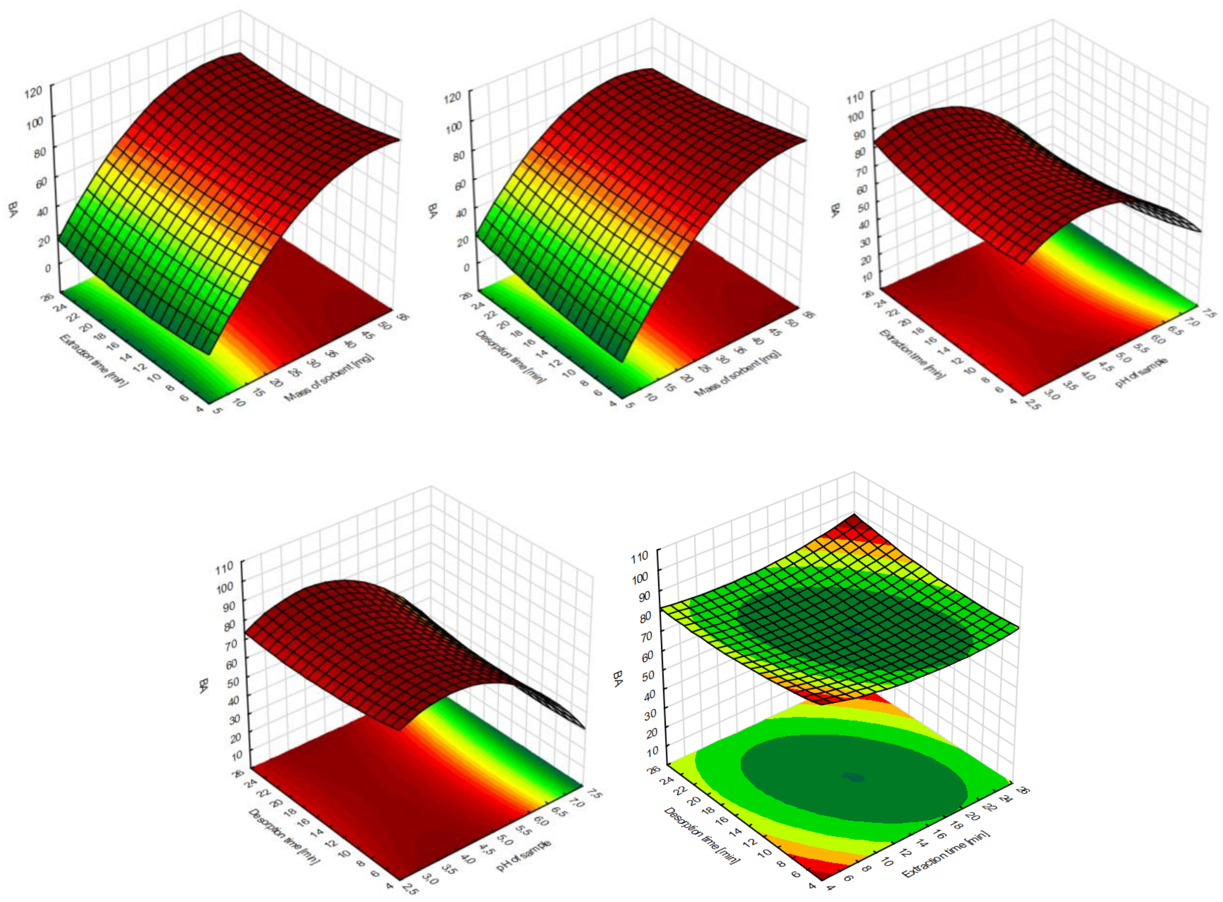

Fig. S3: 3D-graphs of correlation between insignificant parameters for BA:

(1) AxC, (2) AxD, (3) BxC, (4) BxD, (5) CxD.

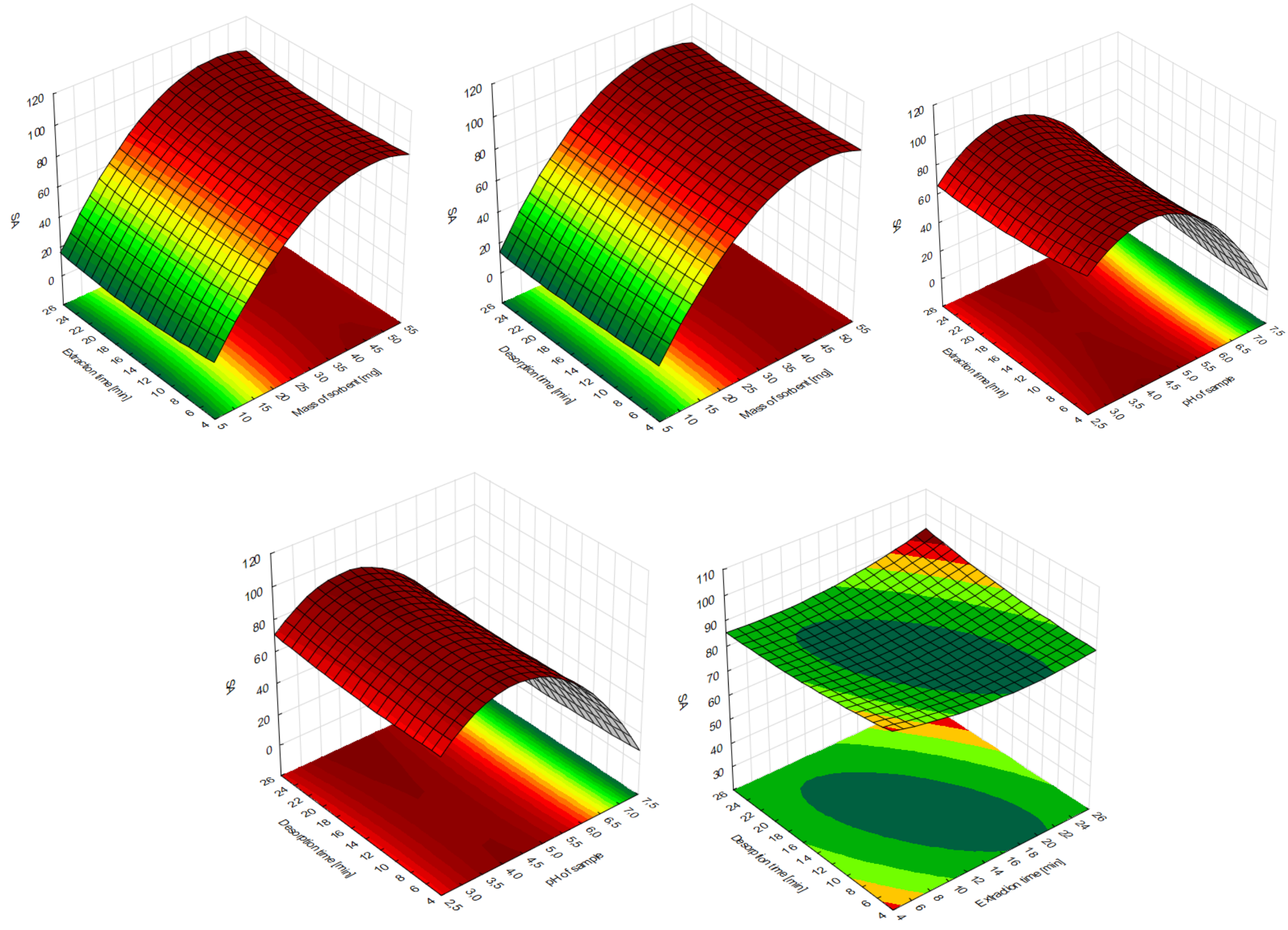

Fig. S4: 3D-graphs of correlation between insignificant parameters for SA:

(1) AxC, (2) AxD, (3) BxC, (4) BxD, (5) CxD.

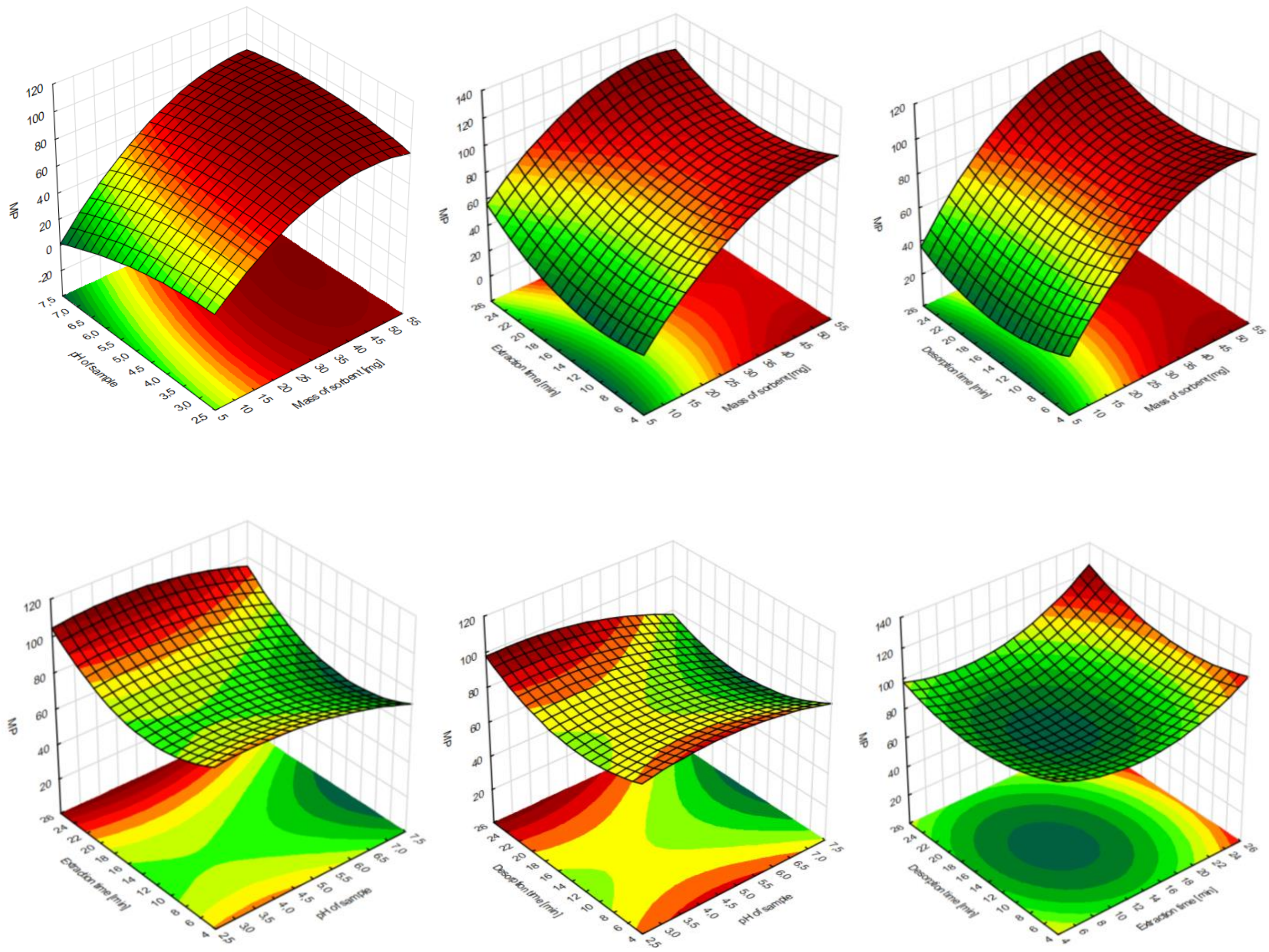

Fig. S5: 3D-graphs of correlation between insignificant parameters for MP: (1) AxB, (2) AxC, (3) AxD, (4) BxC, (5) BxD, (6) CxD.

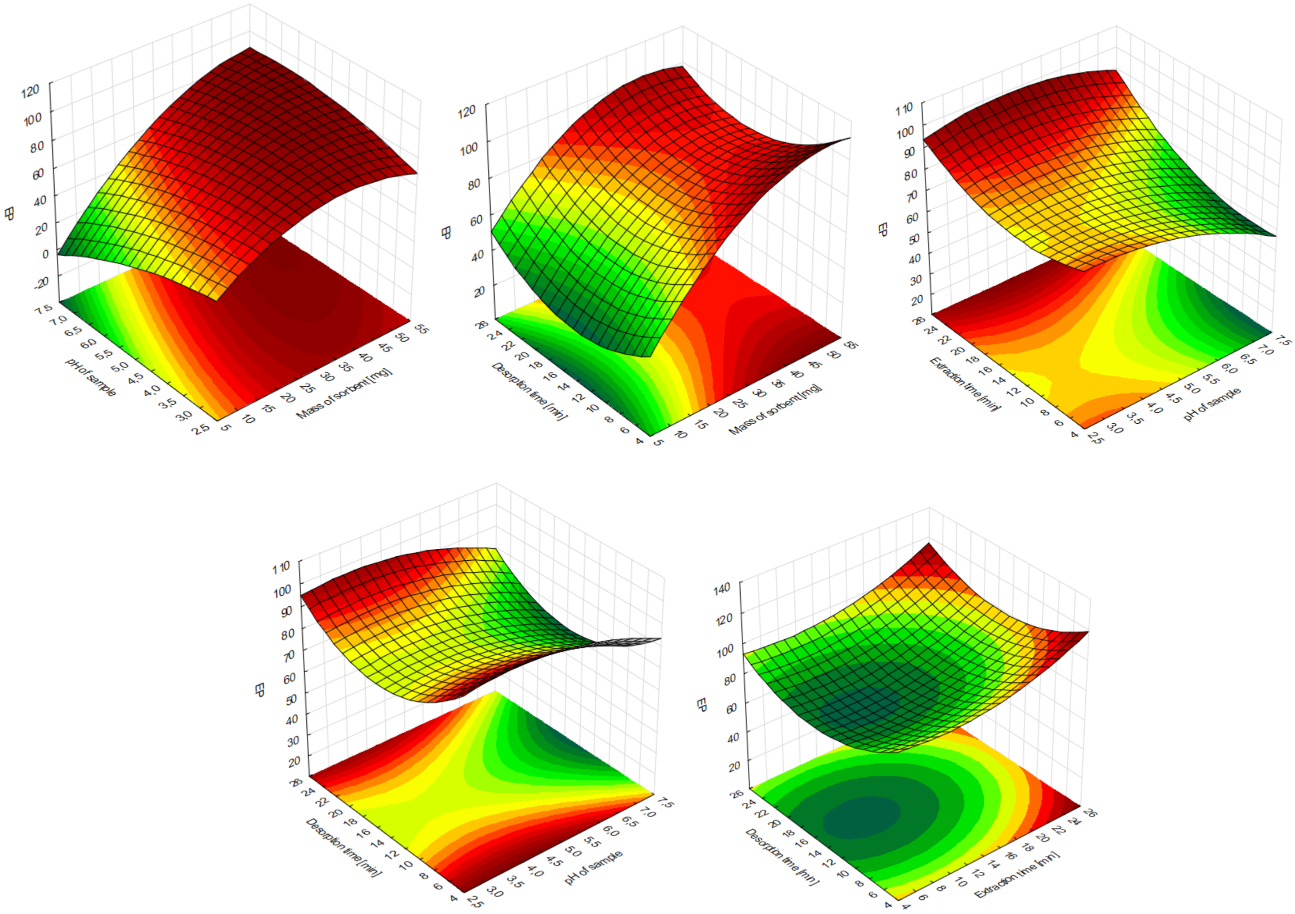

Fig. S6: 3D-graphs of correlation between insignificant parameters for EP: (1) AxB, (2) AxD, (3) BxC, (4) BxD, (5) CxD.

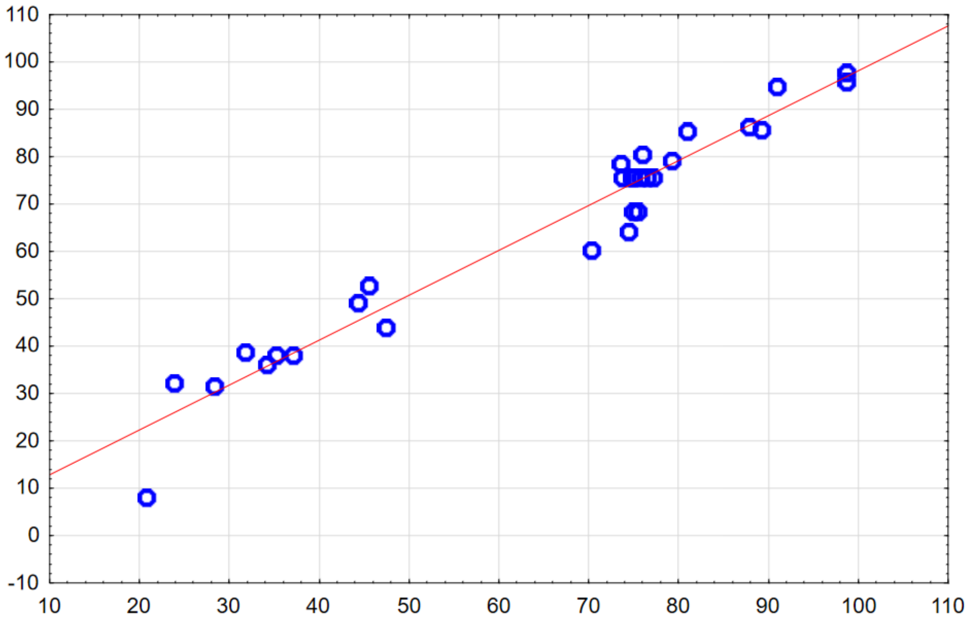

Fig. S7: Correlation between observed and predicted values for ASP.

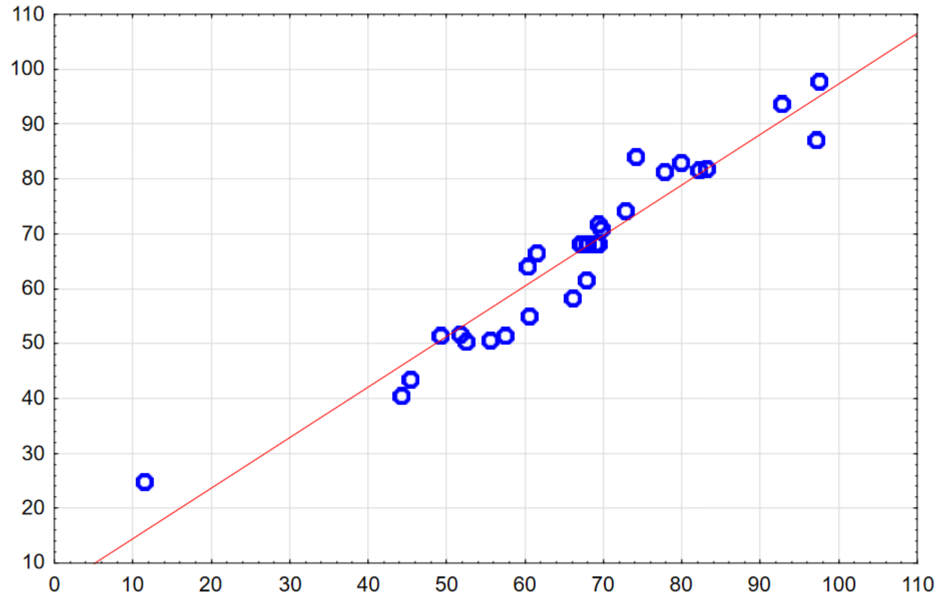

Fig. S8: Correlation between observed and predicted values for ACE-K.

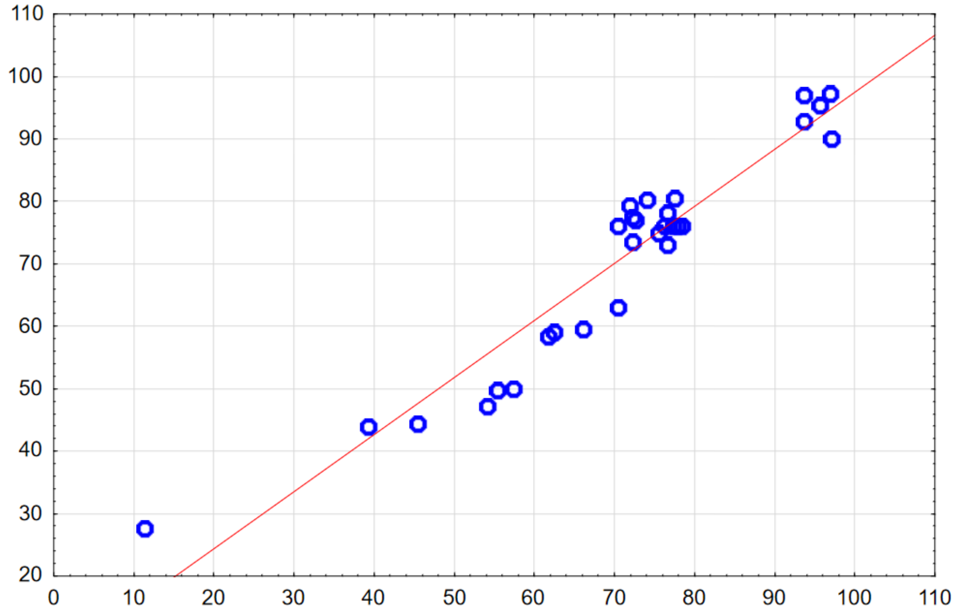

Fig. S9: Correlation between observed and predicted values for BA.

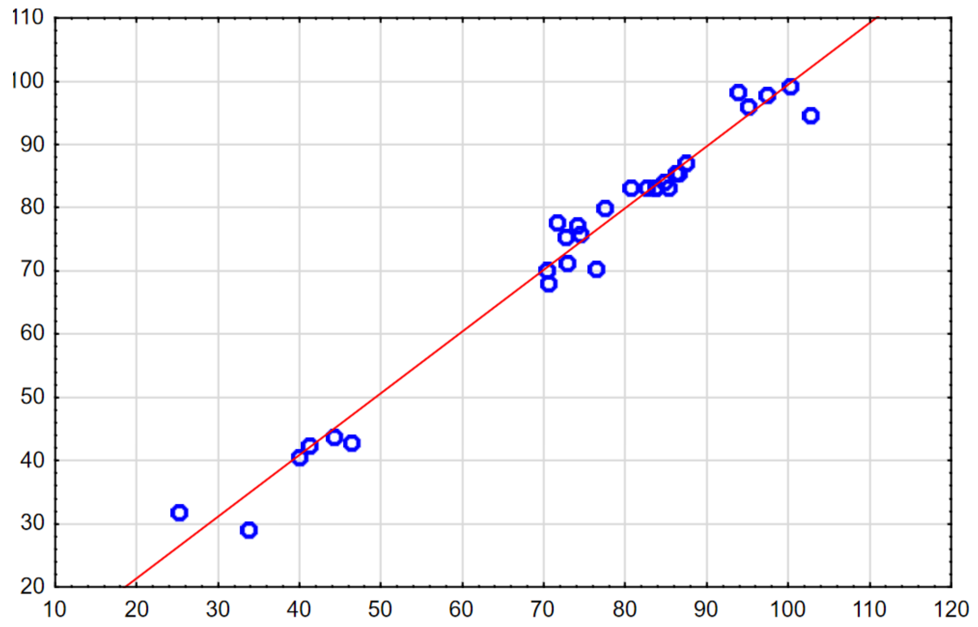

Fig. S10: Correlation between observed and predicted values for SA.

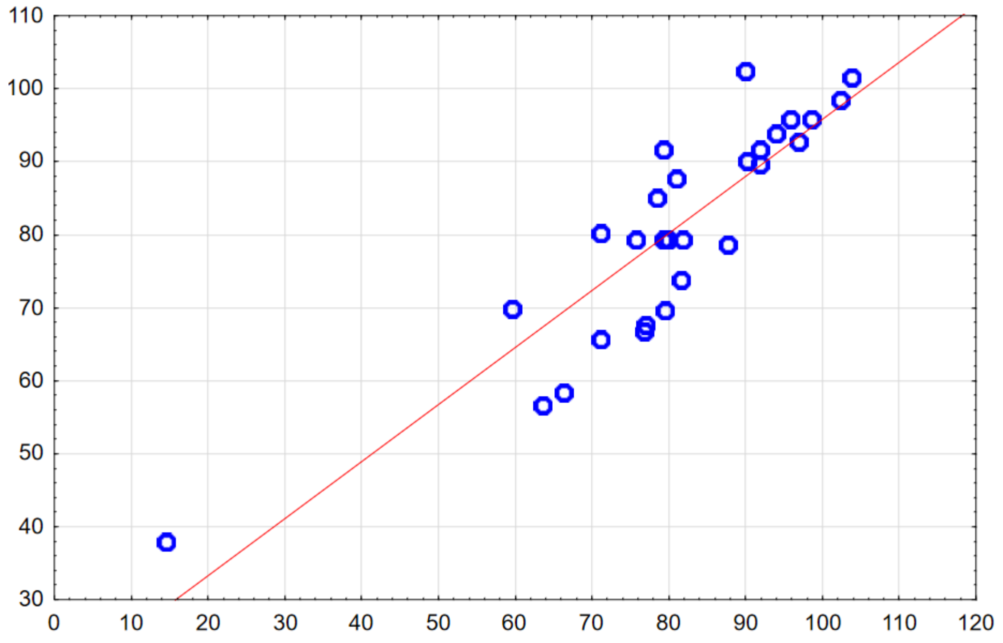

Fig. S11: Correlation between observed and predicted values for MP.

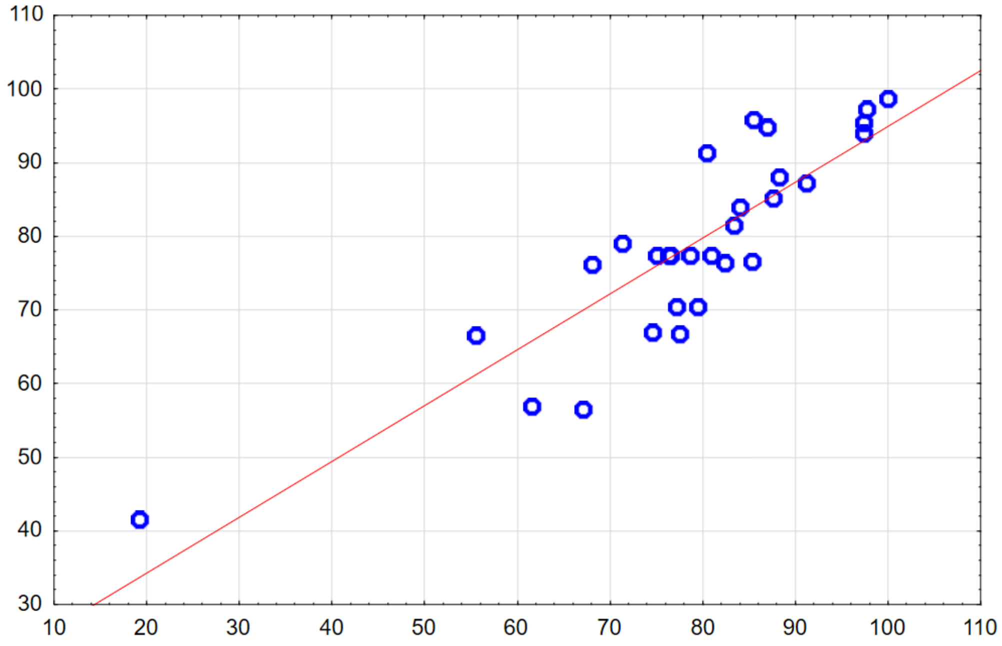

Fig. S12: Correlation between observed and predicted values for EP.

A)

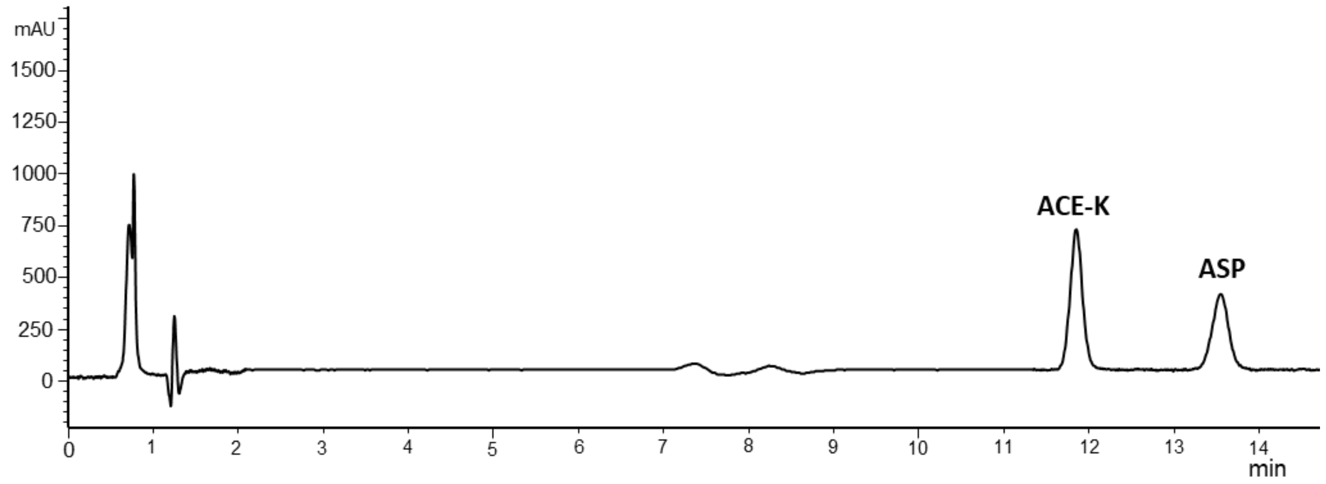

B)

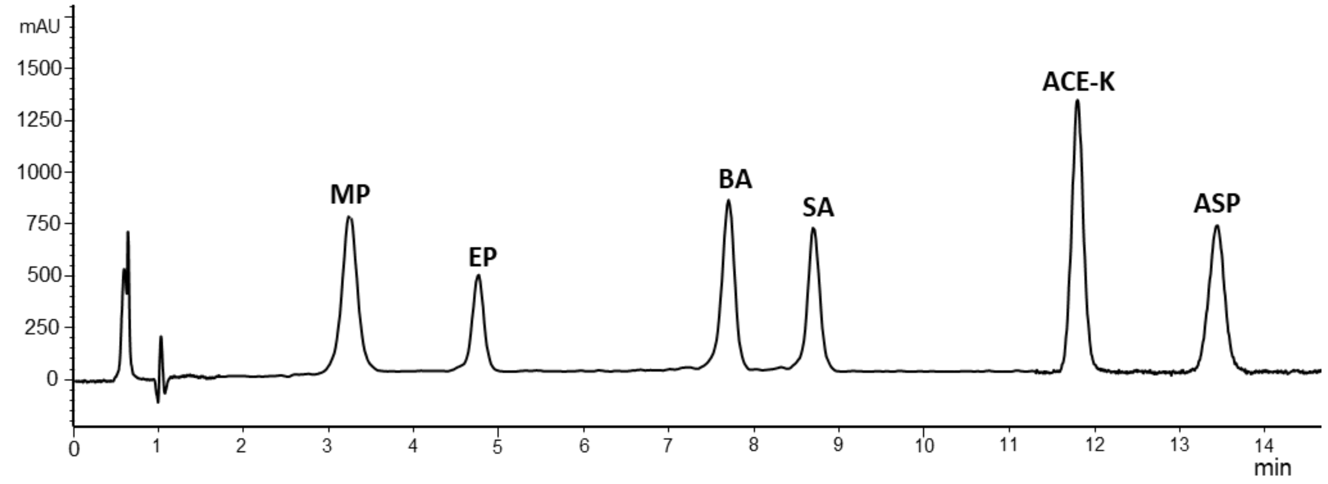

Fig. S13: Chromatograms of isotonic drink type 'light':
A) non-spiked, B) spiked with 50 µg mL^{-1} of each analytes.

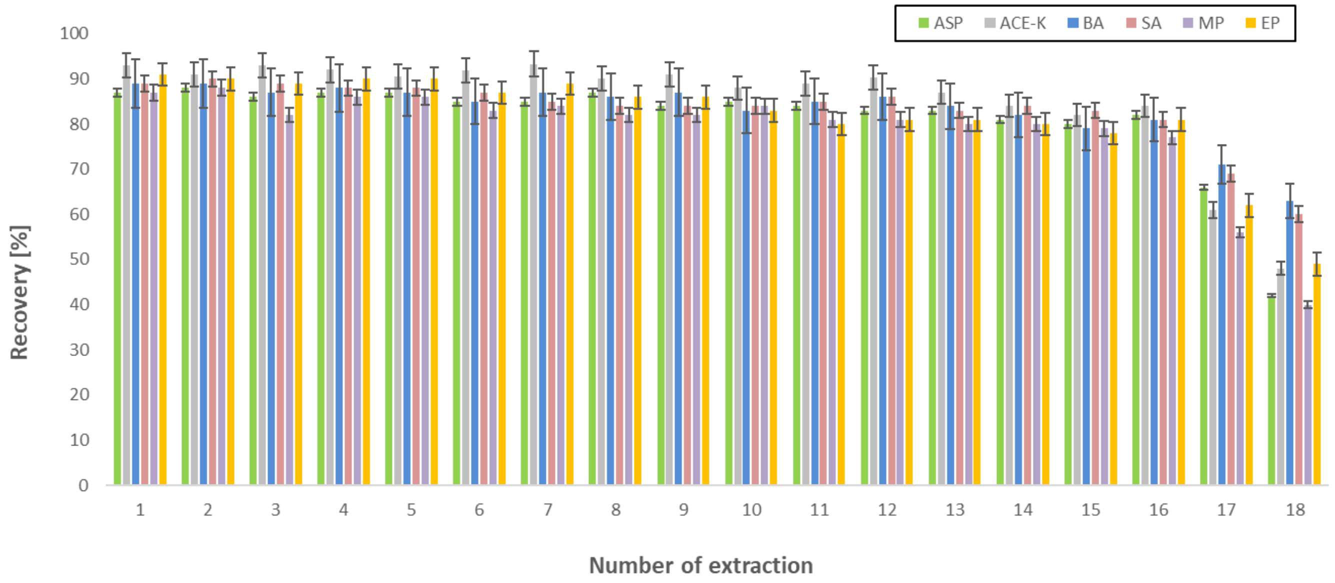

Fig. S14: The chart of the reusability of the AcChCl:DcOH (1:3) for the extraction of ASP, ACE-K, BA, SA, MP and EP.
